# Hemiplegias

**Published:** 1903-02-28

**Authors:** 


### HEMIPLEGIAS.

(*Continued from Page* 352.)

Thrombosis is common in two groups of cases: first, in old age, gout, or diabetes, or convalescence after severe illnesses, such as typhoid and diphtheria, especially when the vessel walls are affected by degenerative changes; and secondly after syphilis and in the neighbourhood of tumours or chronic inflammatory states of the brain. In the first group we have a feeble circulation with probably in most cases some change in the arteries and the blood. In the second the vascular changes may be the sole cause. Paralyses may also be indirectly due to sinus thrombosis either of the infective or marasmic type, the latter occurring in chlorosis, wasting diseases and after typhoid or parturition, and the former chiefly in mastoid disease.

In ordinary arterial thrombosis there are usually premonitory symptoms such as transient aphasia and paralyses, or numbness and tingling in a limb, with insomnia or mental troubles. The stroke itself often comes on gradually for several hours and is rarely accompanied with unconsciousness. It is favoured by feebleness of the circulation and therefore tends to come on during sleep or after a fainting attack or severe fatigue in the aged. It is clear that putting on one side the treatment of the primary disease the precautions needed are those which may prevent cardiac failure, vascular changes, and excessive coagulability of the blood. The treatment of a failing and irregular heart need not be discussed here, except to emphasise the importance of maintaining the horizontal position. We should also warn such patients not to rise and stand up suddenly to pass water, and we should administer to aged and enfeebled persons some stimulating nourishment in the small hours of the night when the circulation is at its lowest. Of course much can be done by the use of the ordinary cardiac tonics, especially those which increase the energy of the heart permanently rather than those, like caffeine which use up the power which it possesses at the time.

Vascular changes have been already touched upon in discussing hæmorrhages, but the changes which arise from malnutrition may sometimes be prevented by administering copious supplies of easily absorbed food as often as the digestive organs can cope with it. The patient who is fed on spurious beef tea, meat extracts and alcohol, or whose digestion is neglected, cannot maintain the nutrition of his arteries, though, of course, in certain toxic diseases, such as phthisis and cancer, nutrition fails in spite of our best efforts.

Then comes the question of the coagulability of the blood. A. E. Wright (*Lancet*, July 5th) has shown how greatly we neglect to watch this factor, and how easy it is to estimate, and perhaps to remove, any excessive tendency to form clots. If normal blood takes five minutes to clot, there is no difficulty whatever in discovering whether our patient has blood which clots in one or two minutes. The process takes less trouble and skill than making a blood count, and, having ascertained the fact, we can treat the condition by giving citric acid and other substances which lower the coagulability. The accidents after the injection of gelatine for aneurysm may, he thinks, be due in some cases to thrombus formation where the increase of coagulability has been carried too far. Indeed, lemon-juice has been given daily, as a routine practice, to typhoid and other convalescents to prevent the danger of thrombosis. The calcium salts contained in a milk diet may increase the tendency to thrombosis in persons disposed to it, just as we give calcium chloride to check hæmorrhage. Yet the presence of the danger cannot be known without a frequent estimation of coagulability, because an excess of calcium may actually lower the tendency, and it is only with a definite percentage of calcium that coagulability is at its highest. In short, it clearly seems a duty in conditions where thrombosis is a danger, especially if premonitory symptoms occur in aged, feeble, convalescent, or parturient cases, to estimate the clotting time of the blood, just as we examine the urine or the heart grounds. We may or may not be able to control the other factors, the vascular and cardiac changes, but we should not neglect the condition of the blood itself, if we wish to avoid cerebral thrombi with their attendant hemiplegia and softening afterwards.

When a thrombus has been formed it may continue to grow, or may be absorbed, or remain unchanged till it becomes organised. Clearly then we must continue the same treatment after a stroke has occurred both as to the vessels, the heart, and the blood itself, with additional measures to prevent the detachment of emboli. Can we aid absorption and phagocytosis ? This is at present doubtful, though carbonate of ammonia has long been given for some such reason. In the syphilitic cases immediate administration of iodide with mercurial inunction may act on the occluded vessel and the collateral ones and restore the circulation.

The heart should be supported, and complete rest enjoined. Free purgation must not be allowed. If the bowels are confined, fluids should be given freely before an aperient is allowed, lest the blood be rendered more inclined to clot, and together with this J. Taylor recommends the use of strychnine, trinitrine, and even of alcohol.

In all forms of hemiplegia rest and care of the digestion are important. After the first day or two gentle passive movements of the limbs should be ordered ; the shoulder especially should be moved freely since it frequently becomes fixed by painful adhesions. Massage of the affected limbs, unless there is advanced renal failure, is next to be employed, and after a month electrical treatment of the paralysed muscles, particularly of the extensors, should be carefully given for a time, and will materially aid the recovery of function. Indeed, with persistent effort, surprising improvement may be made, though less in the case of thrombotic lesions than after embolisms.

